# Influence of interapplicator distance on multibipolar radiofrequency ablation during physiological and interrupted liver perfusion in an in vivo porcine model

**DOI:** 10.1038/s41598-020-71512-x

**Published:** 2020-10-01

**Authors:** F. G. M. Poch, C. A. Neizert, B. Geyer, O. Gemeinhardt, L. Bruder, S. M. Niehues, J. L. Vahldiek, K. K. Bressem, M. E. Kreis, K. S. Lehmann

**Affiliations:** 1 Charité – Universitätsmedizin Berlin, corporate member of Freie Universität Berlin, Humboldt-Universität Zu Berlin, and Berlin Institute of Health, Department of General, Visceral and Vascular Surgery - Campus Benjamin Franklin, Berlin – Hindenburgdamm 30, 12203 Berlin, Germany; 2 Charité – Universitätsmedizin Berlin, corporate member of Freie Universität Berlin, Humboldt-Universität Zu Berlin, and Berlin Institute of Health, Department of Radiology - Campus Benjamin Franklin, Berlin – Germany – Hindenburgdamm 30, 12203 Berlin, Germany

**Keywords:** Preclinical research, Liver cancer

## Abstract

Radiofrequency ablation (RFA) is a curative treatment option for early stage hepatocellular carcinoma (HCC). Vascular inflow occlusion to the liver (Pringle manoeuvre) and multibipolar RFA (mbRFA) represent possibilities to generate large ablations. This study evaluated the impact of different interapplicator distances and a Pringle manoeuvre on ablation area and geometry of mbRFA. 24 mbRFA were planned in porcine livers in vivo. Test series with continuous blood flow had an interapplicator distance of 20 mm and 15 mm, respectively. For a Pringle manoeuvre, interapplicator distance was predefined at 20 mm. After liver dissection, ablation area and geometry were analysed macroscopically and histologically. Confluent and homogenous ablations could be achieved with a Pringle manoeuvre and an interapplicator distance of 15 mm with sustained hepatic blood flow. Ablation geometry was inhomogeneous with an applicator distance of 20 mm with physiological liver perfusion. A Pringle manoeuvre led to a fourfold increase in ablation area in comparison to sustained hepatic blood flow (p < 0.001). Interapplicator distance affects ablation geometry of mbRFA. Strict adherence to the planned applicator distance is advisable under continuous blood flow. The application of a Pringle manoeuvre should be considered when compliance with the interapplicator distance cannot be guaranteed.

## Introduction

Radiofrequency ablation (RFA) has become an established therapy option for treating early stage hepatocellular carcinoma (HCC)^1,2^. RFA is conventionally used in mono- or bipolar mode, where high frequency alternating current is delivered into the liver tissue by one applicator. However, the effective application of RFA is limited to a tumour size of ≤ 3 cm^1^. Furthermore, vascular cooling effects due to the natural liver perfusion (“heat sink effect”) reduce therapy success of RFA^3^. A temporary inflow occlusion of the blood supply to the liver (Pringle manoeuvre) represents a possibility to minimize vascular cooling and increase the maximum ablation area^4–6^. As advantages of a minimally invasive procedure are lost if a Pringle manoeuvre is performed, the application of multibipolar RFA (mbRFA) could also be a viable option to achieve larger ablation areas^7^.

mbRFA represents an advancement to conventional mono- and bipolar RFA techniques. Multiple bipolar electrodes can be used simultaneously so that high-density electrical current flows exclusively between the inserted electrodes^8^. Recently, mbRFA has further been extended to no-touch mbRFA, where direct contact with malignant tissue is avoided by placing multiple electrodes around the tumour. Studies showed that no-touch mbRFA reduces the risk of needle track seeding and moreover, is able to assure an adequate peritumoral safety margin^9–11^. As larger ablation areas are achieved, no-touch mbRFA is also becoming a promising option for the curative treatment of HCC > 5 cm^12–14^. However, interapplicator distance may play an important role in order to generate a sufficient ablation area^15^. Incomplete necrosis seems possible if interapplicator distance is too large, whereas overheating of the ablation centre may occur if the distance is too narrow. To our knowledge, no in vivo study has evaluated the effect of a distance change between the applicators in no-touch mbRFA so far.

The objective of this in vivo animal study was to evaluate the impact of the distance between applicators and the impact of a Pringle manoeuvre on ablation area and geometry in mbRFA. A macroscopic analysis of the ablations was verified by a histological evaluation.

## Results

24 multibipolar radiofrequency ablations were performed in 12 female domestic pigs (weight: 68 ± 11.5 kg; age: 3 months). Three ablations each were possible in two pigs whereas two ablations each were achieved in seven pigs. One mbRFA each was carried out in three pigs. One ablation had to be terminated due to cardiovascular instability of an animal during the application of a Pringle manoeuvre. This ablation was excluded from our analysis. Ablation times were 16:07 min:s [15:08; 22:57] for noPringle20mm, 20:38 min:s [20:02; 21:14] for Pringle20mm and 17:29 min:s [15:46; 21:49] for noPringle15mm. Ablation time was longer for ablations with Pringle manoeuvre in comparison to ablations without Pringle manoeuvre (p = 0.009).

### Qualitative analysis

**Figure 1:**
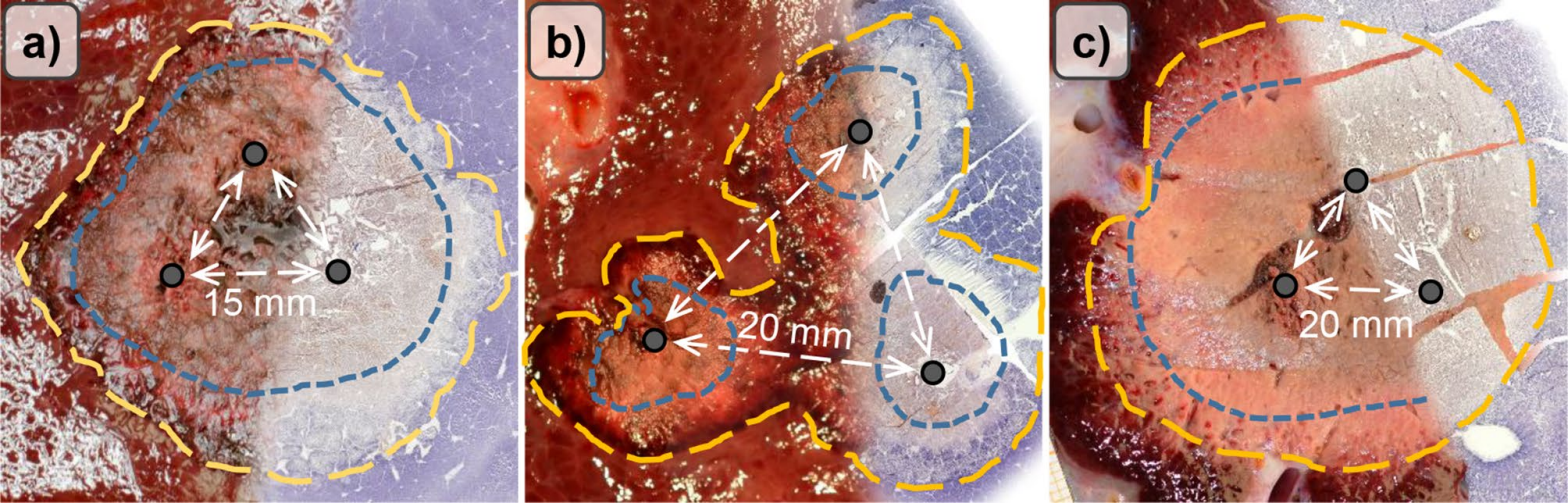
(**a**) Applicator distance 15 mm (noPringle15mm): mainly confluent ablations were seen in this test series. Good correlation was documented between histological (right side) and macroscopic findings (left side). (**b**) Applicator distance 20 mm (noPringle20mm): irregular shaped, two- and three-parted ablations were observed in this test series. While the red zone (dashed, outer line) was mainly confluent, the white zone (dotted, inner line) showed considerable inhomogeneity. Good correlation was seen for macroscopic and histological findings. (**c**) Applicator distance 20 mm with Pringle manoeuvre (Pringle20mm): large and confluent ablations occurred in this test series. The red (mRZ) and white zone (mWZ) could be well distinguished macroscopically, while only one ablation zone (hWZ) was observed histologically.

Typical ablations zones, consisting of mWZ and mRZ, could be observed macroscopically in all three experimental settings (Fig. 1). A histological WZ and RZ were seen in experimental settings with sustained hepatic perfusion (noPringle20mm and noPringle15mm). In contrast to experiments with hepatic perfusion and macroscopic findings, no histological red zone could be identified in ablations with hepatic inflow occlusion (Pringle20mm). In these cases, the white zone directly merged into native liver tissue. No transitional fringe of oedematous tissue was observed around the white zone.

**Figure 2:**
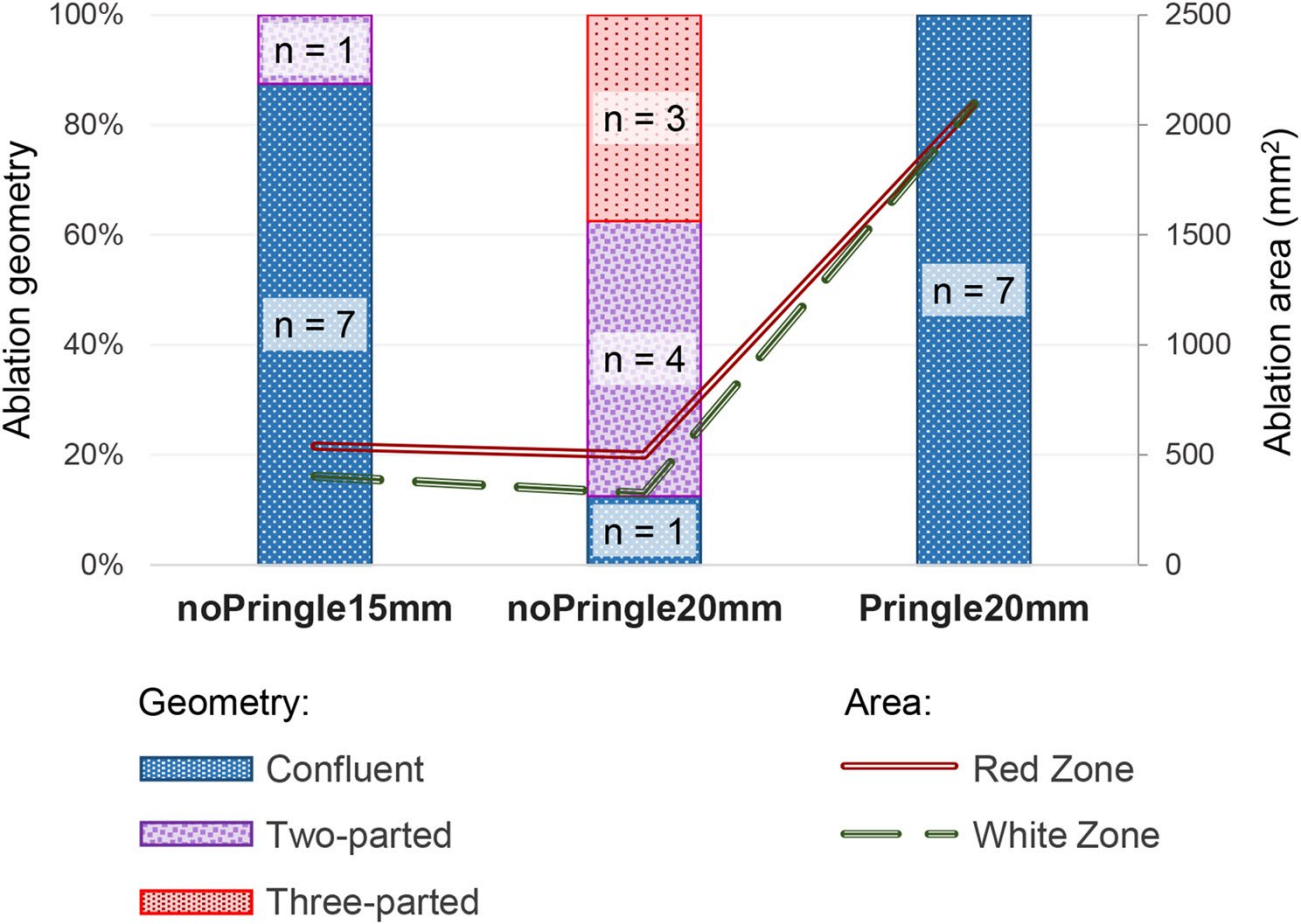
Results of the semi-quantitative analysis of ablation geometries according to Fig. 3. Additionally, ablation area is presented on the right ordinate axis (red and green dotted lines). No change in ablation area could be observed for ablations with hepatic perfusion, while a Pringle manoeuvre resulted in about fourfold larger ablation areas. Ablation geometry was homogenous and confluent in all cases following a Pringle manoeuvre.

Ablation geometry was strongly affected by the experimental set-up. Results of the semi-quantitative analysis are shown in Fig. 2. All Pringle20mm ablations showed one single coherent ablation geometry with no separations. Only one ablation was not confluent in noPrinlge15mm. In this ablation, one of the three applicators was situated within a major hepatic vessel (diameter: 6 mm), which resulted in a two-parted ablation.

### Quantitative analysis

Table 1: Measured ablation areas, radii and RI for macroscopic and histologic findings (median (min.–max.), * p < 0.05; ** p < 0.01). Macroscopic findings were correlated with histological findings in all test series. shows the correlation of macroscopic measurements with a histologic analysis of ablation area, ablation radius and RI. High correlation was found between the mRZ and hRZ in all test series. Poor correlation could be demonstrated between the mWZ and hWZ when a Pringle manoeuvre was performed (Pringle20mm). Histologically only one ablation zone could be detected in this test series. This ablation zone corresponded to the hWZ as only avital cells could be identified. Macroscopic calculations underestimated histological findings in ablations with discontinued hepatic perfusion.

**Table 1:** Measured ablation areas, radii and RI for macroscopic and histologic findings (median [min; max]), * p < 0.05; **p < 0.01).

	noPringle15mm		noPringle20mm		Pringle20mm	
	Macro	Histo	Macro	Histo	Macro	Histo
**Area (mm**^**2**^**)**						
Red zone	554 [413; 627]	540 [394; 603]	494 [234; 638]	500 [153; 630]	2,040 [1,998; 3,034]	2,095 [2,009; 3,317]
*Correlation*^a^	0.83*		0.91**		0.86*	
White zone	400 [303; 497]	403 [293; 454]	321 [194; 462]	324 [126; 457]	1,458 [1,299; 1,689]	2,095 [2,009; 2,403]
*Correlation*^b^	0.88**		0.98*		0.11	
**Maximum radius (mm)**						
Red zone	18 [15; 27]	19 [15; 28]	21 [14; 27]	19 [14; 27]	30 [29; 39]	29 [27; 40]
*Correlation*^a^	0.98**		0.93**		0.82*	
White zone	15 [14; 19]	15 [12; 19]	18 [11; 25]	19 [12; 25]	26 [23; 31]	29 [27; 38]
*Correlation*^b^	0.95**		0.74*		0.04	
**Regularity index (RI)**^c^						
Red zone	0.4	0.4	0.0	0.1	0.6	0.7
White zone	0.4	0.4	0.0	0.0	0.7	0.7

^a,b^Macroscopic findings were correlated with histological findings in regard to the complete ablation area and maximum radius (a), as well as in regard to the ablation area and maximum radius of the white zone (b). Spearman's rank correlation coefficient was applied to analyse the correlation.

^c^A regularity index (RI) defined as ratio between minimum and maximum radius (R_min_/R_max_) was calculated. RI values close to 1.0 are equivalent to a nearly spherical ablation geometry^32^

While applicator distance (15 mm and 20 mm with sustained hepatic blood flow) strongly affected ablation geometry (Fig. 2), applicator distance did not have an impact on complete ablation area (mRZ: 554 mm^2^ (noPringle15mm) vs 494 mm^2^ (noPringle20mm); p = 0.28). Furthermore, a closer applicator distance resulted in an even larger central white zone macroscopically (mWZ: 400 mm^2^ (noPringle15mm) vs 321 mm^2^ (noPringle20mm); p = 0.04). A Pringle manoeuvre led to larger ablations in comparison to ablations with sustained liver perfusion (mRZ: 2,040 mm^2^ (Pringle20mm) vs 554 mm^2^ (noPringle20mm); p < 0.001).

## Discussion

This in vivo animal study demonstrates the importance of an exact positioning of the applicators in mbRFA. While wider applicator distances did not have a major impact on the total ablation area, applicator distance substantially influenced the ablation geometry. Irregular and discontiguous ablations resulted from increasing the applicator distance by a few millimetres from 15 to 20 mm. An increase of ablation area was observed for ablations when a Pringle manoeuvre was performed. These ablations were round and contiguous. The results of our study were validated by a histological analysis of all ablations. We could show close conformity between histological and macroscopic findings in all ablations with sustained liver perfusion. Poor conformity was observed for the macroscopic and histological white zone in ablations with interrupted hepatic perfusion (Pringle manoeuvre).

To our knowledge, there is no study which has investigated the influence of applicator distance in mbRFA in vivo so far. The risk of incomplete tumour ablation in the centre of mbRFA and overtreatment of surrounding healthy liver tissue due to extensive applicator distances has been described as a result of numerical simulation^15^. The effect of applicator distance on mbRFA with 4–6 applicators was analysed in an ex vivo study by *Stoffner* et al^16^. *Stoffner* et al. could demonstrate that larger applicator distances resulted in larger ablation volumes. However, extensive distances between the applicators (> 10 cm) led to insufficient ablations around the respective applicator. Similar results have been described for simultaneous multi-antenna microwave ablation (MWA)^17,18^. Incomplete ablations with indentations were observed when spacing between a total of three antennas exceeded 20 mm^18^. These results indicate that there is a cut off in ablation volume in dependence of the applicator distance. A previous ex vivo study demonstrated that higher energy settings are required to induce round and confluent ablation geometries for larger applicator distances^19^. It seems likely, that this factor is even more relevant in in vivo ablations due to the energy loss caused by the liver perfusion (heat sink effect). This could be confirmed by our in vivo findings. A dose–effect curve in regard to applicator distance and energy input would be highly desirable to make mbRFA in patients more predictable and to avoid incomplete ablation in the “no-touch gap” between the applicators.

The macroscopic results of our study were confirmed by a histological analysis. We could identify a macroscopic and histological red and white zone in the experiments with hepatic perfusion. There was a good correlation between histological and macroscopic findings. This is in accordance with previous ex vivo and in vivo studies for bipolar RFA and MWA^20,21^. Surprisingly, no histological red zone could be identified in all experiments with hepatic inflow occlusion. The histological white zone directly merged into native liver tissue. This is relevant as RFA is primarily assessed through a macroscopic analysis of the WZ in experimental studies. However, the actual area of a Pringle manoeuvre ablation would have been considerably underestimated by a sole macroscopic evaluation without an additional histological verification. So far, no study has described similar results in respect to the missing red zone in mbRFA. Since the grade of cell destruction is defined by tissue temperature, it seems likely that there is a steep drop in temperature at the edge of an ablation. Further research, like generating a temperature map of an ablation with hepatic inflow occlusion, would be desirable to understand this effect.

Noticeable discrepancy between ablation geometries and area could be observed in experiments with obtained hepatic perfusion. This seems worthy of consideration since technical success of minimally invasive procedures is often confirmed by immediate postinterventional contrast-enhanced computed tomography (CECT)^9,13,22^. CECT is not able to distinguish between the red and white zone^23,24^. This may be critical in cases where the white zone is discontinuous, while the red zone is confluent (cf. Fig. 1b). In these cases, CECT would suggest confluent ablations, while vital cells remain in the centre of an ablation. A Pringle manoeuvre should be considered in cases where incomplete ablations are probable. In accordance with other studies, we demonstrated that a Pringle manoeuvre resulted in a fourfold increase in ablation area in comparison to mbRFA without inflow occlusion^23,25^. Treatment success seems more likely if a Pringle manoeuvre is performed. However, there is an increase of damage to adjacent structures^26^. The application of an intermittent Pringle manoeuvre could be an alternative to reduce this risk, while the ablation area remains the same^27^.

This study has some limitations. Firstly, native porcine liver was used in this in vivo study. To our knowledge, there is no existing tumour model for swine. However, tissue changes associated with HCC influence electrical conductivity and resistance in RFA^28^. Therefore, the results of this study should only be considered as an approximation to the ablation areas in real patients. Secondly, animals were euthanized in deep anaesthesia immediately after ablation. This study investigated liver tissue, which was processed immediately after mbRFA. No statement can be made regarding the progression of the ablation zones in the long run. Previous studies reported that parts of the inner red zone decrease in areas, while the outer part of the red zone stays vital^29^. Long-term studies are desirable to investigate changes of the ablation zones over time. Our study was also limited to predetermined ablation parameters. Thus, it is not possible to predict the outcome of ablation geometries in dependence to higher energy input, larger active length of the applicators or different applicator distances. Strict adherence to the preplanned ablation parameters, especially to the applicator distances, can rarely be achieved in a clinical set-up due to anatomical structures like the lung, ribs or vessels. Lastly, Spearman’s rank correlation coefficient was used to test for correlation of ablation area and maximum ablation radius. Close correlation could be observed between histological and macroscopic findings. However, no statement can be made statistically regarding the exact spatial uniformity. However, close spatial uniformity between histological and macroscopic findings was observed visually (Fig. 1). Therefore, additional statistical analysis was waived.

Although, mbRFA seems to be a more complex procedure in comparison to mono- or bipolar RFA, this technique represents a promising therapeutic option in order to overcome some limitations of conventional RFA^9,13^. In regard to local tumour control, mbRFA is even considered superior to other minimally invasive therapies such as MWA used for treating HCC ≤ 5 cm^30^. However, large and more confluent ablations have also been described in experimental studies for MWA using multiple antennas simultaneously^17^. Therefore, direct comparison of clinical efficacy between mbRFA and simultaneous multi-antenna MWA seems relevant to further improve ablative therapy options for HCC.

We could demonstrate that accurate adherence to the applicator distance is essential in order to achieve round and homogenous ablations. Besides applicator length and energy input, applicator distance influences ablation area and geometry in mbRFA. The development of exact dose–effect curves seems mandatory in order to make mbRFA an even more reliable and predictable local ablative procedure.

A strict adherence to the planned applicator distances in mbRFA is highly recommended to achieve round and homogenous ablations. In cases in which an adherence to the preplanned applicator distances cannot be secured, a temporary hepatic inflow occlusion (Pringle manoeuvre) should be considered. Further research evaluating the effect of applicator distances in mbRFA is needed.

## Methods

### Animals and anaesthesia

Female domestic pigs (crossbreed of *Pietrain* and *Topigs*) were obtained from a breeding farm. The pigs were acclimatized for at least ten days before the experiments at 15–24 °C and a day-night cycle of 12 h at the Department of Experimental Medicine, Charité Universitätsmedizin Berlin (certification number ISO 9001:2015). All experiments were performed under general anaesthesia. Animals were anesthetized by an initial intramuscular and subsequent intravenous application of ketamine, azaperon, xylazine and atropine as described previously by *Vahldiek* et al.^23^. Animals received fentanyl as pain medication. A transurethral or suprapubic catheter was placed into the bladder. Vital parameters (i.e. heart rate, oxygen saturation) were monitored throughout the experiments. Access to the peritoneal cavity was achieved by a median laparotomy combined with an incision along the right costal arch to expose the liver. Animals were euthanized in deep anaesthesia by an intravenous injection of T 61 (Embutramid, Intervet Deutschland GmbH, Unterschleißheim, Germany) at the end of the experiments and before hepatectomy.

The protocol of the animal study was approved by the regional office for health and social welfare (LaGeSo, Berlin, Germany, G0281/12). All principles of laboratory animal care were followed according to the guidelines of the European Society of Laboratory Animal Sciences throughout the experiments.

### Radiofrequency ablation

A multipolar ablation system (CELON Power System, Olympus Surgical Technologies Europe, Hamburg, Germany), consisting of a power control unit (CelonLab POWER, Olympus Surgical Technologies Europe, Hamburg, Germany) and a triple peristaltic pump (Celon Aquaflow III, Olympus Surgical Technologies Europe, Hamburg, Germany) that ensured internal cooling of the applicators, was used. A preinstalled RCAP (Resistance Controlled Automatic Power) mode was utilized. Starting power was set to 90 W according to the manufacturers’ recommendations. Energy was delivered by three internally cooled bipolar ablation applicators (CelonProSurge T-30, Olympus Surgical Technologies Europe, Hamburg, Germany) with an active length of 30 mm and a diameter of 1.8 mm. Total energy input was set to 50 kJ.

Three experimental settings (n = 8) were planned with a triangular applicator distance of 20 mm (“noPringle20mm”) and 15 mm (“noPringle15mm”), as well as an applicator distance of 20 mm with hepatic inflow occlusion (Pringle manoeuvre; “Pringle20mm”). A Pringle manoeuvre was performed by temporarily tightening a prepared vessel loop around the hepatoduodenal ligament. Clamping was released immediately after termination of an ablation. A maximum number of possible ablations was performed per animal. Overlap of different ablations was carefully avoided.

**Figure 3:**
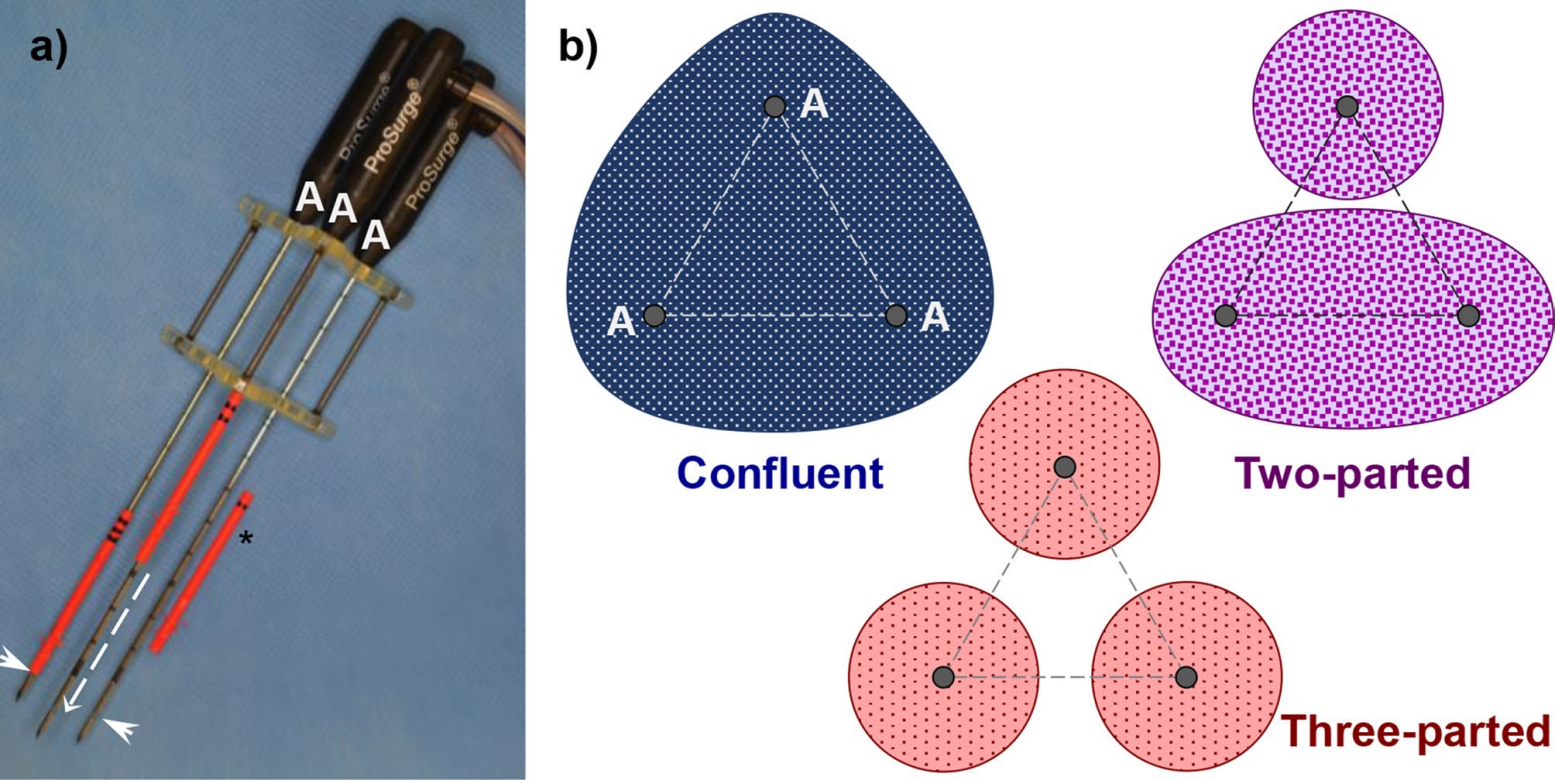
(**a**) Three bipolar RF-applicators were used simultaneously. Exact positioning between the applicators was ensured by a spacer. Applicators were prepared with plastic sleeves (*) prior to an ablation. The plastic sleeves were advanced (dashed arrow) over the applicators after the ablation to mark the maximum cross section of the ablation. The maximum cross section was defined by the insulator situated between the two electrodes on the tip of the applicators (arrowheads). (**b**) Three different ablation geometries are possible in mbRFA. A semi-quantitative analysis was performed according to the following classification: *Confluent*: one single and confluent ablation; *Two-parted:* confluent ablations between two applicators, while the ablation around the third applicator stands alone; *Three-parted:* three isolated, non-confluent ablations around the applicators.

Three plastic tubes with barbs at the tips were placed around the applicator shafts before insertion into the liver (Fig. 3). The tubes were advanced on the applicators towards the centre of the active part of the applicators after an ablation. Subsequently, the applicators were carefully removed. Thus, the tips of the three tubes marked largest cross-section of an ablation. Spacing of the three applicators resulted in a triangular formation. The ablation centre was represented by the geometric centre of the applicator triangle.

### Evaluation of the ablation zones

Ablations were identified and resected and thereafter placed in a custom-made cutting device. Ablations were cut along the largest cross-sectional area marked by the plastic tube and afterwards photographed besides a millimetre scale for subsequent measurements. Ablations were cut into 20 × 30 × 5 mm slices and frozen immediately with liquid nitrogen. 6–8 µm cryosections were cut from the ablation samples with a cryostat (Thermo Scientific CryoStar NX70, Thermo Fisher Scientific, Waltham, USA) and stained for histological examination. A vitality staining with a combined solution of reduced α-NADH (nicotinamide adenine dinucleotide) and NBTC (nitroblue tetrazolium chloride) was used to differentiate between vital and necrotic tissue. Intact liver parenchyma appeared dark blue, while irreparably damaged cells showed white discoloration^31^. Stained cryosections were scanned with a high-resolution scanner (Super Coolscan 5000 ED, Nikon Corporation, Tokyo, Japan; optical resolution 4,000 dpi, colour depth 16 bit, scanning period 38 s).

A semi quantitative analysis was performed in order to describe ablation geometries (Fig. 3). Minimum and maximum radius as well as the cross-sectional area of the so-called “white zone” and “red zone” were determined digitally. The white zone is the area of an ablation, in which immediate cell necrosis occurs^20,32^. This area is macroscopically pale grey. The white zone is adjacent to the outer red zone, in which cell destruction is incomplete and tumour recurrence may occur^33^. Histological findings were marked with “h” (hWZ, hRZ) and macroscopic findings were marked with “m” (mWZ, mRZ). A regularity index (RI) defined as ratio between minimum and maximum radius (R_min_/R_max_) was calculated. RI values close to 1.0 are equivalent to a nearly spherical ablation geometry^32^. Macroscopic findings were correlated with histological findings^20^.

### Statistical analysis

Statistical analysis was performed with a statistical software (IBM SPSS Statistics 25, International Business Machines Corporation, New York, USA). Data are expressed as median and range in brackets [min; max]. The application of a Shapiro–Wilk test showed not normally distributed data. Therefore, the Mann–Whitney U test was used for comparisons between two independent groups, while the Kruskal–Wallis test was used for more than two independent groups. Spearman’s rank correlation coefficient was applied to analyse the correlation between histological and macroscopic findings. Level of significance was 0.05 (two sided) for each statistical testing.

## Data Availability

The datasets analysed in our study are available from the corresponding author on reasonable request.
